# Association of Gender with Clinical Expression, Quality of Life, Disability, and Depression and Anxiety in Patients with Systemic Sclerosis

**DOI:** 10.1371/journal.pone.0017551

**Published:** 2011-03-09

**Authors:** Christelle Nguyen, Alice Bérezné, Thierry Baubet, Caroline Mestre-Stanislas, François Rannou, Agathe Papelard, Sandrine Morell-Dubois, Michel Revel, Loïc Guillevin, Serge Poiraudeau, Luc Mouthon, on behalf of the Groupe Français de Recherche sur la Sclérodermie

**Affiliations:** 1 Université Paris Descartes, Faculté de Médecine Paris Descartes, Pôle de Médecine Interne, Centre de Référence pour les Vascularites Nécrosantes et la Sclérodermie Systémique, Hôpital Cochin, Assistance Publique-Hôpitaux de Paris (AP-HP), Paris, France; 2 Université Paris Descartes, Faculté de Médecine Paris Descartes, Institut Fédératif de Recherche sur le Handicap, Service de Médecine Physique et de Réadaptation, Hôpital Cochin, AP-HP, Paris, France; 3 Université Paris XIII, EA 3413, Service de Psychopathologie, Hôpital Avicenne, AP-HP, Bobigny, France; 4 Université Lille 2, Service de Médecine Interne, Centre de Référence pour la Sclérodermie Systémique, Hôpital Claude Huriez, Lille, France; Marienhospital Herne - University of Bochum, Germany

## Abstract

**Objectives:**

To assess the association of gender with clinical expression, health-related quality of life (HRQoL), disability, and self-reported symptoms of depression and anxiety in patients with systemic sclerosis (SSc).

**Methods:**

SSc patients fulfilling the American College of Rheumatology and/or the Leroy and Medsger criteria were assessed for clinical symptoms, disability, HRQoL, self-reported symptoms of depression and anxiety by specific measurement scales.

**Results:**

Overall, 381 SSc patients (62 males) were included. Mean age and disease duration at the time of evaluation were 55.9 (13.3) and 9.5 (7.8) years, respectively. One-hundred-and-forty-nine (40.4%) patients had diffuse cutaneous SSc (dcSSc). On bivariate analysis, differences were observed between males and females for clinical symptoms and self-reported symptoms of depression and anxiety, however without reaching statistical significance. Indeed, a trend was found for higher body mass index (BMI) (25.0 [4.1] *vs* 23.0 [4.5], p = 0.013), more frequent dcSSc, echocardiography systolic pulmonary artery pressure >35 mmHg and interstitial lung disease in males than females (54.8% *vs* 37.2%, p = 0.010; 24.2% *vs* 10.5%, p = 0.003; and 54.8% *vs* 41.2%, p = 0.048, respectively), whereas calcinosis and self-reported anxiety symptoms tended to be more frequent in females than males (36.0% *vs* 21.4%, p = 0.036, and 62.3% *vs* 43.5%, p = 0.006, respectively). On multivariate analysis, BMI, echocardiography PAP>35 mmHg, and anxiety were the variables most closely associated with gender.

**Conclusions:**

In SSc patients, male gender tends to be associated with diffuse disease and female gender with calcinosis and self-reported symptoms of anxiety. Disease-associated disability and HRQoL were similar in both groups.

## Introduction

Systemic sclerosis (SSc) is a connective-tissue disease characterized by excessive collagen deposition in the dermis and internal organs, and by vascular hyper-reactivity and obliterative microvascular phenomena [1]. SSc is responsible for diminished life expectancy, related to skin extent and visceral involvement [2]. SSc is also responsible for tendon, joint, and vessel damage, leading to disability, handicap, and impaired health-related quality of life (HRQoL) [3]. In addition, psychiatric symptoms, including anxiety and depression, have been reported as a consequence of disease chronicity in SSc patients, with a prevalence of depressive symptoms ranging from 18% to 65% [4], [5], [6], [7], [8], [9], [10], [11], [12].

Consistent with other auto-immune diseases, SSc is predominant among females, with a ratio of females to males of 1∶1 to 14∶1 [13], along with gender differences in disease activity and incidence. Such differences have been explained by genetic and hormonal factors and lifestyle [14], [15]. Male gender is usually considered a factor of poor prognosis in SSc [16], [17]. A cohort of 91 SSc patients (10% males) from Spain revealed clinical and immunological differences between the genders; arthralgias were more often encountered in females, whereas myositis and nucleolar antinuclear antibodies were more frequent in males [15]. More recently, as compared with female SSc patients, males were found to more often exhibit renal failure, increased blood pressure, arrythmia and inflammatory myopathy and less often sicca syndrome and anti-centromere antibodies. Causes of death and mortality also differed between the sexes [18]. In a large European cohort of 1180 patients with early SSc (19% males), features of diffuse disease were significantly more frequent in males [19]. Recently, Hudson *et al.* found that the time to diagnosis was longer for women than men after the onset of Raynaud's phenomenon, and suggested that there may be possible biologic differences in the progression of disease or in the health care trajectories of men and women with early SSc [20].

Although gender differences in disease-related clinical manifestations are well established, few studies have compared HRQoL, disability, and psychiatric symptoms between male and female patients with SSc. In the present study, we aimed to assess the association of gender with clinical expression, HRQoL, disability, and self-reported symptoms of depression and anxiety in patients with SSc.

## Methods

### Study design

We performed a cross-sectional survey of 381 patients. Patients with SSc were prospectively included during 7 consecutive annual meetings of the French SSc patients' association, the “Association des Sclérodermiques de France” (ASF), between 2003 and 2009, or during their hospitalization in the internal medicine departments of Cochin (between January 2006 and June 2009) or Claude Huriez (between January and June 2009) hospitals. Since some patients were evaluated during several ASF annual meetings, only the most recent assessment of each patient was considered. Patients had to complete self-administered questionnaires first and then to undergo an interview with a physician to check for unanswered question, fully complete questionnaires, and gather clinical data.

### Patients

To be eligible for the study, patients had to fulfil the American College of Rheumatology [21] and/or the Leroy and Medsger [22] criteria for SSc. Patients from the ASF were assessed within 48 hr during spring (temperature 20°C). Parameters recorded were age; sex; age at disease onset; disease duration; body mass index (BMI); disease subset (limited SSc [lSSc], limited cutaneous SSc [lcSSc] or diffuse cutaneous SSc [dcSSc]); mouth opening (inter-incisor distance measured in millimetres); skin involvement; telangiectasia; Raynaud's phenomenon; pitting scars; digital ulcers; calcinosis; gastrointestinal tract, joint and/or muscle involvement; dyspnoea (assessed by the New York Heart Association [NYHA] 4-point scale); ILD; echocardiography systolic pulmonary artery pressure [PAP]>35 mmHg); and renal crisis. History of esophagus, gastrointestinal, joint, muscle and/or heart involvement; ILD; echocardiography PAP>35 mmHg; and renal crisis was obtained from detailed clinical charts for hospitalized patients and self-reports for ASF members.

### Health status

Health status was assessed by the KPS score, the scale ranging from 0 (dead) to 100 (normal no complaints; no evidence of disease) [23]. Originally developed for cancer patients, because it strongly predicted cancer outcome [24], [25], the KPS score has been shown to provide clinical estimates of patient's physical state, performance, and prognosis and to be associated with social status in patients with SSc [23], [26].

### Health-related quality of life

HRQol was assessed by the French version of the Medical Outcomes Study 36-Item Short Form Health Survey (SF-36) [27], a self-administered questionnaire covering 8 areas: physical function, physical role, bodily pain, general health, vitality, social function, emotional role, and mental health. For each area, scores range from 0 (poorer health status) to 100 (better health status). Scores can also be summarized in 2 global scores: physical component score (PCS) and mental component score (MCS).

### Disability

#### Global disability

Global disability was assessed by use of the standard disability index of the Health Assessment Questionnaire (HAQ-DI) that contains 20 items (each scored ranging from 0 [no disability] to 3 [maximal disability]), divided into 8 domains [28].

#### Patients' perceived disability

Patients' perceived disability was assessed by the McMaster Toronto Arthritis Patient Preference Disability Questionnaire (MACTAR) [29]. Patients were asked to select the 3 situations among activities of daily living (ADL) that caused them maximal trouble [23]. Each item is scored on an 11-point quantitative scale (range 0–10). The global score ranges from 0 (no disability) to 30 (maximal disability). This score has been validated in SSc [23], [30].

#### Hand disability

Hand disability was assessed by the Cochin Hand Function Scale (CHFS) [31], a questionnaire administered by the physician that contains 18 items related to ADL. Each question is scored on a scale of 0 (performed without difficulty) to 5 (impossible to do). The total score is obtained by adding the scores of all items (range 0–90). This questionnaire has been validated in SSc [32].

#### Mouth disability

Mouth disability was assessed by the Mouth Handicap In Systemic Sclerosis (MHISS) scale, a questionnaire administered by the physician that contains 12 items concerning difficulties in performing ADL. Each question is scored on a scale of 0 (never) to 4 (always) [33]. The total score is obtained by adding the scores of all items (range 0–48).

### Anxiety and depression

Self-reported anxiety and depression symptoms were assessed by the Hospital Anxiety and Depression scale (HADS). This scale has 7 questions for the anxiety dimension (HADa) and 7 for the depression dimension (HADd) [34]. Each item is scored on a scale of 0 to 3, the total score ranging from 0 (no depression, no anxiety) to 21 (maximal depression, maximal anxiety). Scores of 0–7 in subscales are considered normal, 8–10 borderline and ≥11 pathologic cases [35]. The definition of clinical anxiety and/or depression was based on the HADS score cutoff ≥8 found to be relevant in patients with autoimmune diseases [36].

### Aesthetic impairment

Aesthetic impairment was assessed on an 11-point quantitative scale, the total score ranging from 0 (no aesthetic impairment) to 10 (maximal aesthetic impairment).

### Statistical analysis

Data analysis involved use of Systat 9 (SPSS Inc., Chicago, IL, USA). Quantitative variables were described with means ± standard deviations (SD) and qualitative variables with frequencies and percentages. For bivariate analysis, parametric tests were used since all parameters met criteria for normal distribution. Comparisons between male and female groups involved the Pearson chi-square test for qualitative variables and two-sample *t* test for quantitative data. Bonferroni adjustment was used for multiple comparisons (43 comparisons); therefore a *p* value less than 0.001 was considered statistically significant. Multivariate analysis was used to determine the association of gender and SSc-related variables. Backward stepwise regression all-inclusive analysis was run, including all dependent variables, with values of 0.20 to enter and 0.10 to stay in the model. Adjustment for age and type of recruitment from either patient association or hospitalization was performed. Odds ratios (OR) and 95% confidence intervals were calculated.

### Ethics statement

This survey was conducted in compliance with the protocol of Good Clinical Practices and Declaration of Helsinki principles. Patients gave their consent to participate after being orally informed about the study protocol. In accordance with European regulation (Directive 2001/20/EC of the European Parliament and of the Council of 4 April 2001 on the approximation of the laws, regulations and administrative provisions of the Member States relating to the implementation of good clinical practice in the conduct of clinical trials on medicinal products for human use; Directive 95/46/EC of the European Parliament and of the Council of 24 October 1995 on the protection of individuals with regard to the processing of personal data and on the free movement of such data), French observational studies from data obtained without any additional therapy or monitoring procedure, do not need formal approval of an Institutional Review Board or an Independent Ethics Committee, and a formal written consent from the patients is not required for this kind of project.

## Results

### Demographic and clinical data

Overall, 381 patients were included. One-hundred-and-forty-three of them were recruited during their hospitalization in the internal medicine departments of Cochin (n = 127) or Claude Huriez (n = 16) hospitals, and the remaining 238 patients were recruited during ASF annual meetings from 2003 to 2009. The proportion of patients from the ASF who agreed among those who were asked to participate were 51 among 80 (63.8%) (44 females) in 2003, 50 among 80 (62.5%) (44 females) in 2004, 71 among 98 (72.4%) (59 females) in 2005, 70 among 95 (73.7%) (55 females) in 2006, 70 among 101 (69.3%) (55 females) in 2007, 86 among 130 (66.1%) (74 females) in 2008 and 2009 alltogether. Of the 381 patients, 62 were males (16.4%), with a female to male ratio of 5∶1. The mean age at the time of evaluation was 55.9 (13.3) years, and mean disease duration was 9.5 (7.8) years. A total of 149 (40.4%) patients had dcSSc, 187 (50.7%) had lcSSc, and 34 (9.2%) had lSSc. The mean KPS was 77.6 [11.7] (range 50–100) (Table 1).

**Table 1:** Demographic and clinical characteristics of patients with SSc*.

Age, years, mean (SD)	55.9 (13.3)
Age at disease onset, years, mean (SD)	46.2 (12.9)
Male sex	62/379 (16.4)
Patient association	62/191 (32.5)
Disease duration, years, mean (SD)	9.5 (7.8)
Body mass index, kg/m^2^, mean (SD)	23.4 (4.5)
Diffuse cutaneous SSc	149/369 (40.4)
Limited cutaneous SSc	187/369 (50.7)
Limited SSc	34/369 (9.2)
KPS (0–100), mean (SD)	77.6 (11.7)
Inter-incisor distance, mm, mean (SD)	35.9 (9.3)
Skin involvement	339/370 (91.6)
Telangiectasia	253/347 (72.9)
Raynaud's phenomenon	369/377 (97.9)
Pitting scars	221/376 (58.8)
Digital ulcers	170/375 (45.3)
Calcinosis	105/312 (33.7)
Gastrointestinal tract involvement	304/375 (81.1)
Arthralgia	254/375 (67.7)
Myalgia	209/375 (55.7)
Dyspnea, NYHA classification, mean (SD)	2.1 (0.8)
Interstitial lung disease	163/373 (43.7)
Echocardiography systolic PAP>35 mmHg	48/375 (12.8)
Scleroderma renal crisis	34/375 (9.1)

*Values are number/number of patients for whom the data is available (%), otherwise indicated in parenthesis.

KPS: Karnofsky performance status; NYHA: New York Heart Association; PAP: pulmonary artery pressure; SD: standard deviation; SSc: systemic sclerosis.

### Association of gender with SSc clinical expression

Males and females were comparable in age at the time of evaluation and at disease onset, disease duration and health status as assessed by the KPS score. For other clinical variables, some differences were observed between males and females, without reaching statistical significance. Indeed, BMI was higher in males than females (25.0 [4.1] *vs* 23.0 [4.5], p = 0.013) (Table 2). DcSSc was more frequent in males (54.8 *vs* 37.2%, p = 0.010), whereas lSSc was more frequent in females (10.7% *vs* 1.6%, p = 0.024). Regarding visceral involvement, males more often exhibited ILD and echocardiography PAP>35 mmHg than did females (54.8% *vs* 41.2%, p = 0.048; and 24.2% *vs* 10.5%, p = 0.003, respectively), and females more often calcinosis than males (36.0% *vs* 21.4%, p = 0.036). On multivariate logistic regression, gender was significantly associated with BMI (OR 1.12, 95% confidence interval [CI] 1.01–1.24) and echocardiography PAP>35 mmHg (OR 0.23, 95% CI 0.07–0.76).

**Table 2:** Association of gender with clinical manifestations in SSc*.

	Malesn = 62	Femalesn = 319	p-value^†^
Age, years (mean [SD])	55.7 (14.5)	55.9 (13.1)	0.924
Age at disease onset, years (mean [SD])	46.7 (14.4)	46.1 (12.7)	0.749
Disease duration, years (mean [SD])	8.9 (6.8)	9.7 (8.1)	0.577
Body mass index, kg/m^2^ (mean [SD])	25.0 (4.1)	23.0 (4.5)	0.013
Diffuse cutaneous SSc	34/62 (54.8)	115/309 (37.2)	0.010
Limited cutaneous SSc	27/62 (43.5)	160/309 (51.8)	0.237
Limited SSc	1/62 (1.6)	33/309 (10.7)	0.024
KPS (0–100) (mean [SD])	78.0 (11.6)	77.5 (11.7)	0.755
Inter-incisor distance, mm (mean [SD])	37.0 (9.4)	35.7 (9.3)	0.359
Skin involvement	59/62 (95.2)	280/310 (90.3)	0.221
Telangiectasias	46/60 (76.7)	207/289 (71.6)	0.426
Raynaud's phenomenon	59/62 (95.2)	310/317 (97.8)	0.237
Pitting scars	41/62 (66.1)	180/316 (57.0)	0.180
Digital ulcers	28/61 (45.9)	142/316 (44.9)	0.890
Calcinosis	12/56 (21.4)	93/258 (36.0)	0.036
Gastrointestinal tract involvement	50/62 (80.6)	254/315 (80.6)	0.999
Arthralgias	39/62 (62.9)	215/315 (68.3)	0.411
Myalgias	32/62 (51.6)	177/315 (56.2)	0.507
Dyspnea, NYHA classification (mean [SD])	2.2 (0.8)	2.0 (0.9)	0.190
Interstitial lung disease	34/62 (54.8)	129/313 (41.2)	0.048
Echocardiography systolic PAP>35 mmHg	15/62 (24.2)	33/315 (10.5)	0.003
Scleroderma renal crisis	8/62 (12.9)	26/315 (8.3)	0.243

*Values are number/number of patients for whom the data is available (%), otherwise indicated in parenthesis.

SSc: systemic sclerosis; SD: standard deviation; KPS: Karnofsky Performance Status; NYHA: New York Heart Association; PAP: pulmonary artery pressure.

### Association of gender with SSc HRQoL and disability

HRQol assessed by the SF-36 was comparable in both groups. PCS and MCS were similar, and lower than 40 out of 100, for both males and females (34.2 [10.0] and 35.9 [9.6], p = 0.240, and 35.1 [12.3] and 34.3 [13.0], p = 0.667, respectively). Consistently, regarding global, patient-perceived, and location-specific disability as assessed by the HAQ, MACTAR, and CHFS and MHISS, respectively, we found no gender differences within each of these variables. Both groups exhibited similar aesthetic impairment (Table 3).

**Table 3:** Association of gender with health-related quality of life and disability in SSc*.

	All patientsn = 381	Malesn = 62	Femalesn = 319	p-value^†^
KPS (0–100)	77.6 (11.7)	78.0 (11.6)	77.5 (11.7)	0.755
SF-36 (0–100)				
• Physical functioning	35.7 (24.8)	31.4 (24.7)	36.6 (24.7)	0.152
• Physical role	19.4 (24.8)	15.8 (29.5)	20.1 (31.5)	0.350
• Bodily pain	22.5 (25.7)	19.6 (24.3)	23.1 (25.9)	0.349
• General health perception	24.1 (20.2)	23.9 (22.4)	24.1 (19.8)	0.932
• Vitality	21.9 (20.7)	21.7 (22.6)	21.9 (20.3)	0.973
• Social functioning	30.0 (32.5)	30.4 (34.3)	30.0 (32.2)	0.923
• Emotional role	24.2 (36.5)	24.5 (38.0)	24.1 (36.3)	0.945
• Mental health	32.6 (25.1)	31.6 (27.5)	32.8 (24.7)	0.753
• PCS	34.9 (14.6)	34.2 (10.0)	35.9 (9.6)	0.240
• MCS	38.5 (36.9)	35.1 (12.3)	34.3 (13.0)	0.667
HAQ (0–3)	1.1 (0.8)	1.0 (0.8)	1.1 (0.8)	0.237
MACTAR (0–30)	18.5 (8.2)	18.8 (7.1)	18.4 (8.4)	0.753
CHFS (0–90)	20.1 (19.3)	19.8 (20.3)	20.2 (19.2)	0.890
MHISS (0–48)	19.0 (11.6)	20.4 (15.0)	18.7 (10.7)	0.512
Aesthetic impairment	4.6 (2.6)	4.5 (3.4)	4.6 (2.4)	0.833

* Values are the mean (standard deviation).

SSc: systemic sclerosis; KPS: Karnofsky Performance Status Scale; SF-36: Medical Outcomes Study 36-Item Short Form Health Survey; PCS: Physical Component Score; MCS: Mental Component Score; HAQ: Health Assessment Questionnaire; MACTAR: McMaster-Toronto Arthritis Patient Preference Disability Questionnaire; CHFS: Cochin Hand Function Scale; MHISS: Mouth Handicap In Systemic Sclerosis Scale.

### Association of gender with SSc self-reported symptoms of depression and anxiety

On bivariate analysis, some differences were observed between males and females for self-reported symptoms of depression and anxiety, but without reaching statistical significance. Indeed, self-reported symptoms of anxiety, as defined by HADa subscale score ≥8 were more frequent in females than males (62.3% *vs* 43.5%, p = 0.006), whereas absence of self-reported symptoms of both depression and anxiety, as defined by HADa and HADd subscale scores <8 was more often encountered in males than females (46.8 *vs* 31.6%, p = 0.021) (Table 4). Males and females did not differ in depression symptoms. On multivariate analysis, gender was associated with anxiety only (OR 5.50, 95% CI 1.12–27.04).

**Table 4:** Association of gender with depression and anxiety in SSc*.

	All patientsn = 381	Malesn = 62	Femalesn = 319	p-value^†^
HADa (0–21) (mean [SD])	9.2 (4.5)	8.3 (5.1)	9.4 (4.4)	0.088
• HADa≥8	224/378 (59.3)	27/62 (43.5)	197/316 (62.3)	0.006
HADd (0–21) (mean [SD])	6.6 (4.2)	6.5 (4.6)	6.6 (4.1)	0.781
• HADd≥8	154/378 (40.7)	25/62 (40.3)	129/316 (40.8)	0.957
• HADa and HADd<8	129/378 (34.1)	29/62 (46.8)	100/316 (31.6)	0.021

*Values are number/number of patients for whom the data is available (%), otherwise indicated in parenthesis.

SSc: systemic sclerosis; n: number; HADa: Hospital Anxiety and Depression scale for Anxiety; HADd: Hospital Anxiety and Depression scale for Depression.

## Discussion

In the present study of 381 patients with SSc, we found a ratio of females to males of 5 to 1, which is in agreement with previous studies [14]. Some differences were observed between males and females for clinical symptoms and self-reported symptoms of depression and anxiety, however without reaching statistical significance. Indeed, dcSSc, echocardiography PAP>35 mmHg and ILD were more often encountered in males, whereas lSSc and calcinosis were more often encountered in females. Females were more frequently found with self-reported symptoms of anxiety. Conversely, we found no association with gender regarding perceived health status, HRQoL and reported global and location-specific disability. On multivariate analysis, BMI, echocardiography PAP>35 mmHg, and anxiety were the variables most closely associated with gender.

The prevalence of dcSSc in male patients was high and reached 54.8%. DcSSc was more frequent than lcSSc in males. The exact opposite was observed in females and was more consistent with previous reports of epidemiology studies of both male and female SSc patients. In two large US and German studies, the prevalence of lcSSc and dcSSc among SSc patients was 66.2% and 33.8%, and 45.5% and 32.7%, respectively [37], [38]. In 3 cohorts of 1,012 Italian, 249 Swedish and 185 Canadian patients, dcSSc was more frequent in males than females (range from 37% to 67%) [39], [40], [41]. Conversely, 2 studies from Spain comparing male and female SSc patients found no gender differences by disease type [15], [18]. Finally, from a recent retrospective French survey of 121 SSc patients, dcSSc was more frequent in males than in females (22% *vs* 5%) [42]. In these last 3 studies, the male sample sizes were rather small (n = 9, n = 26 and n = 36, respectively).

We found BMI significantly lower in females than in males. This finding might be of clinical relevance despite lack of clear explanatory reports. Indeed, in a prospective multiethnic cohort of 250 SSc patients, low BMI was among the 7 independent variables predictive of mortality. The authors even hypothesized that strong association of low BMI with mortality could be an objective and/or complete surrogate for generalized deconditioning or gastro-intestinal involvement [43].

Females were also more likely to have calcinosis, which is a frequent manifestation of SSc and found in about 25% of patients [44]. Calcinosis mainly affects the extremities, at sites of recurrent microtrauma such as the forearms, elbows or fingers [45]. It occurs predominantly at a late stage of disease and is not restricted to patients with the lSSc [46]. However, clinical features associated with calcinosis remain poorly described. Recently, we provided evidence that calcinosis is an independent factor associated with digital ulcers (OR 2.33, 95% CI 1.04–5.19) [47].

In agreement with previous studies, we found that men were more likely than women to have echocardiography PAP>35 mmHg and ILD, for prevalences of 24.2% and 54.8%, respectively. Lung involvement is common in the course of SSc, and together, ILD and pulmonary hypertension are considered the 2 main causes of death in this disease [48]. ILD is more frequent in male SSc patients at the time of diagnosis and during follow-up [42]. In addition, male gender is associated with pulmonary hypertension during follow-up [42]. In 1180 SSc patients (19% men) studied at early stages of the disease, men more often than women were found to have lung fibrosis and lower diffusing lung capacity for carbon monoxide than women [19]. Thus, lung involvement in male SSc patients requires special attention and specific care because of its frequency and association with poor prognosis.

Interestingly, we found gender differences for both self-reported symptoms of depression and anxiety in SSc. Females more often exhibited self-reported symptoms of anxiety, whereas men were more often free of self-reported symptoms of both anxiety and depression. In a recent cross-sectional survey of 108 patients visiting a rheumatology outpatient department, the only factor significantly associated with psychiatric symptoms was gender [49]. Conversely, in another cross-sectional study of 111 patients visiting a rheumatology clinic, 9% with SSc, gender had no effect on the frequency of anxiety and depression [50]. Finally, in a study designed to assess psychological adjustment of 112 patients with early polyarthritis, female gender was found to be associated with high levels of depression and anxiety [51]. Substantial evidence indicates that females report greater fear and are more likely to have anxiety disorders than are males. Complex processes underlie gender differences in anxiety. Individual differences in etiological factors of anxiety and fear are moderated by socialization processes that prescribe gender-specific expectations for expression of anxiety and the acceptable means of coping with anxiety [52]. Finally, we found no differences in depression symptoms by gender (40.3 *vs* 40.8%, p = 0.96), which is consistent with recent findings by Thombs *et al* [12].

Remarkably, despite our finding of gender differences in clinical expression in SSc, males and females experienced comparable loss of function, global and location-specific disability, and HRQoL impairment, as evidenced by similar HAQ, CHFS, MHISS, MACTAR and SF-36 scores. Gender may not be a major determinant of perceived disability and impaired HRQoL in patients with SSc, and functional and social issues should be considered as severe in males as in females. Consistently, we recently found in a cohort of 87 SSc that employment status was strongly associated with perceived disability and health status but not with gender [26]. In addition, using the World Health Association Disability Assessment Schedule II to assess HRQoL, Hudson *et al* found that clinical correlates of HRQoL did not include gender [53]. HRQoL and functional disability may be associated with the meaning that SSc patients ascribe to their condition, which may be comparable for both males and females, rather than with its severity or its organ manifestation.

Our work has some limitations. Our sample of males was small, and our inability to demonstrate statistically significant differences between the two groups might be due to the lack of statistical power. Another limitation was the procedure used to recruit patients. Since all patients belonged to the French association of patients or were hospitalized in tertiary care units, they may not be representative of the whole French SSc population. Patients had longstanding disease, which could imply more symptoms. HAQ scores were high but remained comparable to those reported from previous studies conducted in tertiary care settings [54]. Moreover, patients recruited from the patient association may have had more severe SSc than hospitalized patients [55]. Further studies conducted in other cohorts are required to confirm the gender differences we observed. Finally, our study was not designed to explore the reasons for the observed gender differences. One can only hypothesize about the associated etiological factors, which may involve hormonal influences; genetics such as X-chromosome inactivation and monosomy, or microchimerism; as well as lifestyle (e.g., the debated connection with silicone implants) [14].

In conclusion, we confirm the association of gender and clinical manifestations in patients with SSc. Diffuse disease and lung involvement are more frequent in males, whereas females more often exhibit calcinosis and self-reported symptoms of anxiety. Despite SSc patients displaying gender-related clinical differences, the disease impact on perceived health status, HRQol and disability, is comparable in both groups. Studies comparing male and female patients living in different countries, with different occupational, lifestyle and medical exposure, would also be of interest to further clarify the role of environmental factors in such gender differences.
